# A Global Perspective of Childhood Vaccinations: Past, Present, and Future—From Smallpox to mRNA

**DOI:** 10.1111/apa.70677

**Published:** 2026-07-15

**Authors:** Tobias Alfvén, Tania Reza, Alieu Sowe

**Affiliations:** ^1^ Department of Global Public Health Karolinska Institutet Stockholm Sweden; ^2^ Sachs' Children and Youth Hospital Stockholm Sweden; ^3^ Ministry of Health Banjul The Gambia

## Abstract

Childhood immunisation is one of the most successful public health interventions in history, preventing millions of deaths and substantially reducing global child mortality. This mini‐review summarises the historical development of childhood vaccines, the role of global coordination in expanding immunisation coverage, and the innovations that have enabled safe and effective vaccine delivery to children worldwide. It also examines persistent and emerging challenges, including inequities, limited manufacturing capacity, vaccination hesitancy, and climate‐related disruptions. Finally, the review highlights opportunities in the current vaccine pipeline and the importance of sustained global collaboration to ensure equitable protection for all children.

## Summary

- Childhood immunisation has dramatically reduced child mortality, but progress remains uneven, and many children are still not reached by life‐saving vaccines.
- Global coordination, technological innovation, and strong safety systems have driven major successes, while inequities, vaccination hesitancy, and climate‐related disruptions threaten future gains.
- Sustained investment, resilient health systems, and equitable access to new vaccines are essential to further improve child survival and health equity.

## 1 Introduction

Immunisation is widely considered one of the best investments in public health, with programmes targeting children yielding particularly substantial returns. In the past 50 years, paediatric vaccines are estimated to have averted approximately 154 million deaths—the majority among children under five—and reduced global infant mortality by 40% [1]. Each dollar invested in childhood immunisation returns nearly fifty‐fold in health and economic benefits [2]. However, gains are unevenly distributed and remain vulnerable to complex and interconnected factors that threaten to reverse hard‐won progress [3]. Understanding the history of immunisation, the factors that have enabled its success, and the challenges that threaten its future is essential to ensuring all children are protected against preventable disease. This mini‐review provides a brief overview of the history and contemporary landscape of childhood vaccination with a focus on low‐ and middle‐income countries.

## 2 Historical Foundations

The origins of vaccination can be traced to efforts to combat smallpox, a disease with estimated case fatality rates of 8%–10% for endemic smallpox, but in fully susceptible populations ranging between 45% and 55%, and up to 80% among infants [4, 5]. Edward Jenner's 1796 observation that exposure to cowpox conferred immunity to smallpox marked the beginning of the modern vaccination era. Nearly a century later, Louis Pasteur developed the first laboratory‐made vaccine using an attenuated bacterium [6], which, together with subsequent advances in inactivated and toxoid technologies, facilitated the development of vaccines against tuberculosis, diphtheria, tetanus, pertussis, polio, and measles [7, 8, 9]. These vaccines formed the core of early childhood immunisation programmes. A critical innovation came in the late 1940s, when Leslie Collier developed the freeze‐dried smallpox vaccine, overcoming cold‐chain barriers that had previously limited vaccine delivery to children in remote and resource‐limited settings [10]. A summary of key milestones in childhood vaccinations is provided in Figure 1.

**FIGURE 1:**
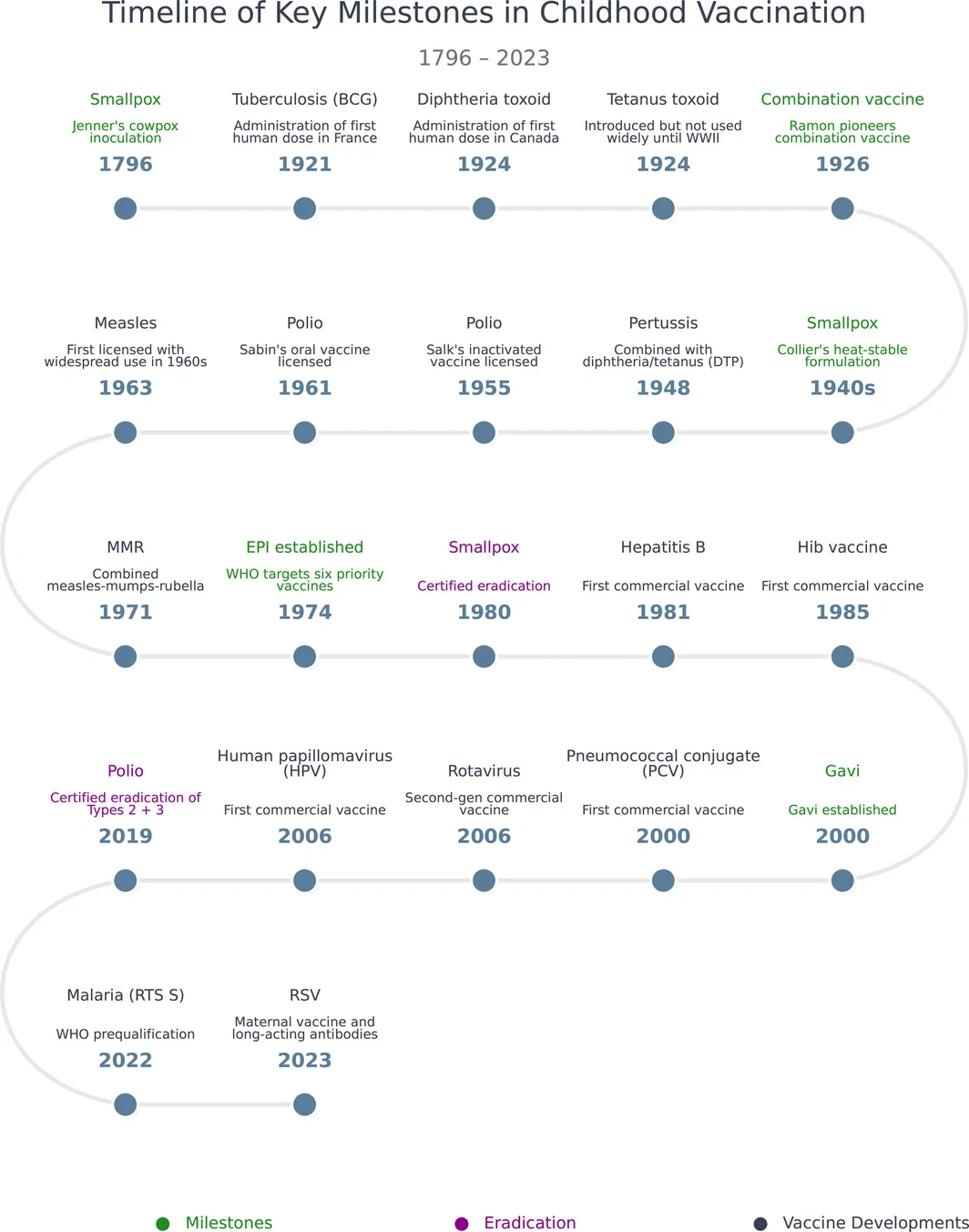
Timeline of Key Milestones in Childhood Vaccination.

## 3 Global Coordination and Key Achievements

Despite advances in vaccine development and delivery, immunisation coverage remained heterogeneous across countries. By the early 1970s, robust immunisation programmes in high‐income countries had expanded routine childhood immunisation schedules to include the newly developed polio vaccine and the measles, mumps, and rubella (MMR) vaccine. In contrast, less than 5% of children in low‐ and middle‐income countries (LMICs) had received their country's core childhood vaccine series [11]. These disparities led to the establishment of the World Health Organisation's Expanded Programme on Immunisation (EPI) in 1974, which initially targeted six diseases (tuberculosis, diphtheria, tetanus, pertussis, polio, and measles) responsible for most vaccine‐preventable deaths in children. In the decade following its creation, EPI increased coverage of these core vaccines from 5% to over 80% in many LMICs, sometimes surpassing coverage in high‐income countries [11].

Vaccine coverage stalled in the 1990s [12], leaving millions of children unreached by immunisation efforts, particularly in conflict‐affected areas, displaced communities, or informal settlements. In response, Gavi, the Vaccine Alliance, was established in 2000 as a public‐private partnership—funded through direct contributions and innovative financing mechanisms—to address vaccine coverage gaps in low‐income countries eligible based on Gross National Income per capita thresholds [13]. Supported countries are expected to co‐finance vaccine procurement at increasing levels and eventually transition off Gavi support entirely [13]. Through this model, Gavi has supported the vaccination of over one billion children in 78 lower‐income countries since its inception and helped avert an estimated 17 million future deaths [14].

The impact of coordinated efforts on child survival has been profound. The eradication of smallpox, certified by WHO in 1980, demonstrated the feasibility of large‐scale immunisation and was made possible by significant investments in health infrastructure, cold chain systems, and health workers [15]. EPI‐supported immunisation campaigns for measles have significantly reduced global case incidence and mortality, with deaths declining by 88% between 2000 and 2024 [16]. Indeed, recent modelling estimates show that most vaccine averted child mortality and morbidity can be attributed to widespread measles vaccination [1]. The Global Polio Eradication Initiative has achieved certified eradication of wild poliovirus Types 2 and 3 in 2015 and 2019, though the Type I serotype remains endemic in Pakistan and Afghanistan [17, 18].

Country‐level achievements further illustrate what coordinated action can accomplish. Nepal eliminated rubella in 2025 following four national vaccination campaigns targeting children between 9 months and 15 years [19]. Uganda witnessed near elimination of *Haemophilus influenzae* type b meningitis (Hib meningitis) – a leading cause of bacterial meningitis in children under 5 with a mortality rate of up to 20%– just seven years after vaccine introduction [20].

## 4 Enabling Factors: Innovation & Safety

Advances in childhood immunisation depend on technological innovation and robust systems to ensure vaccine safety. Combination vaccines have improved timeliness and acceptability by reducing the number of injections children receive [21, 22]. Sri Lanka made significant strides in hepatitis B control, with zero cases of hepatitis B reported in 2023 among children over 5 years old—an achievement the country attributes to high coverage of the pentavalent vaccine [23]. More recently, innovations in monoclonal antibodies, mRNA, and viral vector platforms have improved the speed, immunogenicity, and production capacities of immunisation technologies [24]. These platforms are now being used to develop vaccines against diseases such as respiratory syncytial virus (RSV), malaria, and tuberculosis that continue to kill hundreds of thousands of children annually [25, 26, 27].

Equally important is sustaining public confidence in vaccines, which is primarily achieved through rigorous safety monitoring. Regulatory safeguards, including ethical review, clinical trials, and post‐licensure surveillance, facilitate early detection of safety risks during vaccine development and rollout. Global pharmacovigilance systems, such as the WHO's VigiBase, provide continuous monitoring of medicines and vaccines across 170 countries, including paediatric populations [27]. The history of the rotavirus vaccine illustrates the importance of such safeguards. Despite the efficacy of the first rotavirus vaccine developed in 1998, it was soon withdrawn from the market after post‐licensure surveillance detected a clinically significant risk of intussusception among vaccinated infants who were at least three months old [28, 29]. The next generation of rotavirus vaccines, developed in 2006, was shown to be safe and effective in reducing rotavirus‐associated hospitalisations and deaths in children [28]. Today, they are included in national immunisation programmes around the world. The two most common rotavirus vaccines are first administered at 6 weeks of age, with subsequent doses given to complete the series by 24 or 32 weeks of age, depending on the vaccine. However, WHO guidelines recommend that missed doses be administered up to 24 months of age [28, 30, 31].

## 5 Persistent Challenges to Immunisation

Global immunisation efforts are projected to prevent 5.8 million deaths annually by 2030 [32]. However, progress is threatened by structural inequalities, limited vaccine manufacturing capacity, rising vaccination hesitancy, and climate change.

### 5.1 Structural Inequalities

Differences in health system capacity and financing between high‐income settings and low‐income settings remain central drivers of global vaccine inequity. Limited fiscal space, weaker health systems, and constrained cold‐chain and delivery infrastructure continue to reduce coverage and delay vaccine introduction, particularly for newer and more costly vaccines [33]. These structural disparities are reinforced by global vaccine markets and governance arrangements that favour countries with purchasing power, domestic manufacturing capacity, and regulatory influence [34].

During global health emergencies, such as the COVID‐19 pandemic, these imbalances became especially visible, with high‐income countries securing early vaccine supplies while many low‐income countries depended on delayed donations and redistribution mechanisms [35]. Beyond access to new vaccines, fragile health systems in low‐resource settings are more vulnerable to disruption [36]. During periods of crisis, routine childhood immunisation services are often deprioritised or interrupted, leading to declines in coverage and increases in vaccine‐preventable morbidity and mortality [36, 37]. Similar patterns have been observed in other outbreaks, including mpox, where disease burden and deaths were concentrated among children and adolescents in resource‐limited settings [38]. Demographic factors, such as maternal education, socioeconomic status, and geographic location, are also associated with vaccine uptake [39, 40, 41, 42]. Maternal education and socioeconomic status impact health literacy, vaccination hesitancy, and trust in health systems [40], while socioeconomic status has been shown to affect healthcare access [42]. Unless structural inequities in financing, manufacturing, health system resilience, and access are addressed, global immunisation gaps will persist. Without sustained investment and more equitable governance, children in LICs will continue to bear a disproportionate burden of vaccine‐preventable disease—both during global crises and in routine immunisation programmes.

### 5.2 Vaccine Manufacturing

Limited manufacturing capacity and infrastructure in LMICs create reliance on imported vaccines, often leading to insufficient and unpredictable supply during high‐demand periods, such as the H1N1 pandemic in 2009 [43]. Similarly, newer paediatric vaccines are often introduced in LMICs years after licensure and remain prohibitively expensive for routine use [44]. Addressing these gaps will require sustained investment in technology transfer, regulatory capacity, and sustainable procurement mechanisms to ensure children everywhere have timely access to new and existing life‐saving vaccines.

### 5.3 Vaccination Hesitancy

Even when vaccines are accessible, demand‐side barriers to vaccination leave children vulnerable to preventable diseases. The rapid spread of misinformation through digital platforms can undermine trust in vaccines, even in communities with historically high uptake. The WHO declared vaccination hesitancy—driven by misinformation, politicisation, and fear amplified during the COVID‐19 pandemic—a critical threat that could reverse decades of progress in global health, with declines in the coverage of key vaccines observed since the start of the pandemic [45, 46]. In the United States, e.g., misinformation circulating on social media and the politicisation of routine public health interventions contributed to declining rates among American children, with measles cases in the US exceeding 2000 cases for the first time since 1992 [47, 48]. Investment in health promotion and communications strategies is essential to sustain gains in child vaccine coverage. These strategies must include transparent communication, meaningful community engagement, and application of behavioural and social science insights. Health workers, consistently the most trusted source of vaccine information, need strong support, training, and resources to address parental concerns effectively.

### 5.4 Climate Change

Climate‐driven disasters pose direct threats to immunisation infrastructure. In Pakistan, unprecedented flooding in 2022 ravaged 90 districts across the country, disrupting child immunisation services and damaging 450 health facilities, including cold chain equipment essential for vaccine storage [49, 50]. Immunisation rates have historically decreased during extreme weather events, with the most significant decline among girls and children living in rural areas [51, 52]. Strengthening health system resilience to climate shocks is essential to maintaining immunisation coverage among the world's most vulnerable children.

## 6 The Vaccine Pipeline Opportunities for Child Health

Scientific innovation is advancing at an unprecedented pace, with promise for diseases that disproportionately affect children. Recent strides have been made in the prevention of respiratory syncytial virus (RSV), a leading cause of infant hospitalisation in high‐income countries and death in low‐income countries. As of 2025, the WHO recommends either long‐acting monoclonal antibodies or maternal vaccination during pregnancy, both of which have been shown to prevent RSV infection among infants [53, 54, 55].

Malaria, which kills one child nearly every two minutes [56], has long been considered one of the most challenging targets for vaccine development due to the parasite's complex life cycle and ability to evade immune responses. The recent WHO prequalification of two malaria vaccine candidates, RTS, S and R21, represents a historic milestone, with initial reports suggesting a reduction of 13% in all‐cause child mortality in countries where the RTS, S vaccine was introduced [57]. Second‐generation mRNA‐based candidates aim to further improve durability and efficacy [58]. Tuberculosis remains a leading infectious cause of death among children, and the current BCG vaccine provides limited protection against pulmonary disease. The M72/AS01E vaccine candidate, which demonstrated 54% efficacy in a Phase 2b trial, is now in Phase 3 testing [59]. Success would transform TB prevention in children. Vaccines against Group B Streptococcus, a major cause of neonatal sepsis and meningitis, are also progressing through development pipelines and could prevent substantial morbidity and mortality among children under five.

Ensuring these innovations reach children everywhere will require strong global collaboration, adequate financing, and regulatory harmonisation. If these conditions are met, the next generation of vaccines could deliver major gains in child survival and health equity.

## 7 Global Strategies & Partnerships

The challenges and opportunities outlined above are addressed through an evolving architecture of global partnerships and strategies. Since 2005, the World Health Organisation has led the development of three successive global strategies (1): the Global Immunisation Vision and Strategy (2005–2015), which aimed to expand coverage and integrate immunisation with routine child health services [60] (2); the Global Vaccine Action Plan (2011–2020), which introduced formal monitoring frameworks and addressed financing, equity, and affordability [61]; and (3) the current Immunisation Agenda 2030, which emphasises reaching zero‐dose children and building resilient health systems [62]. These strategies have been operationalised through complementary partnerships.

The Coalition for Epidemic Preparedness Innovations (CEPI) is a partnership established in 2017 between governments, the private sector, philanthropic organisations, NGOs, and civil society [63]. Funded through investments from national governments, the European Commission, and philanthropic foundations, CEPI advances vaccine research and development for emerging infectious diseases, particularly those with pandemic potential. The rapid development of these vaccines—including paediatric formulations where appropriate—will protect children directly and indirectly by reducing community transmission and minimising disruption to routine childhood immunisation services. The World Intellectual Property Organisation (WIPO), a specialised agency of the United Nations system, shapes the intellectual property and technology transfer frameworks that influence vaccine development, production, and distribution [64]. For children in LMICs, technology transfer arrangements and affordable licensing are essential to ensuring timely access to new vaccines. UNICEF's contributions to childhood immunisation include research and development, contractual agreements, and policy frameworks, but their primary roles are supporting governments in administering vaccines and serving as the world's largest single buyer of vaccines for LMICs [65].

## 8 Conclusion

From Jenner's foundational experiments to contemporary mRNA platforms, the history of childhood immunisation represents one of the most consequential achievements in global health. The establishment of EPI, the creation of Gavi, and successive global strategies have brought life‐saving vaccines to billions of children who would have otherwise been left unprotected. Yet sustained progress will depend on addressing persistent equity gaps, building public trust, strengthening vaccine manufacturing resilience, and preparing immunisation systems for the impacts of climate change—threats that will most significantly impact children in low‐resource settings.

At the same time, scientific innovation presents unprecedented opportunities. New and improved vaccines against malaria, tuberculosis, RSV, and Group B Streptococcus could prevent hundreds of thousands of child deaths annually if developed and delivered equitably. Whether these opportunities are realised will depend on sustained political commitment, adequate financing, and cooperation among governments, multilaterals, academia, business, and civil society organisations.

## Author Contributions

**Tobias Alfvén:** conceptualization, writing – original draft, supervision, funding acquisition, methodology, writing – review and editing, project administration. **Alieu Sowe:** writing – review and editing. **Tania Reza:** writing – review and editing, visualization.

## Funding

The authors have nothing to report.

## Conflicts of Interest

The authors declare no conflicts of interest.

## Data Availability

Data sharing not applicable to this article as no datasets were generated or analysed during the current study.
